# HspB1 Overexpression Improves Life Span and Stress Resistance in an Invertebrate Model

**DOI:** 10.1093/gerona/glab296

**Published:** 2021-10-05

**Authors:** Courtney Carroll Alexander, Erin Munkáscy, Haven Tillmon, Tamara Fraker, Jessica Scheirer, Deborah Holstein, Damian Lozano, Maruf Khan, Tali Gidalevitz, James D Lechleiter, Alfred L Fisher, Habil Zare, Karl A Rodriguez

**Affiliations:** 1 Sam and Ann Barshop Center for Longevity and Aging Studies, University of Texas Health Science Center San Antonio (UTHSCSA), San Antonio, Texas, USA; 2 Department of Cell Systems and Anatomy, UTHSCSA, San Antonio, Texas, USA; 3 University of North Carolina at Pembroke, Pembroke, North Carolina, USA; 4 Department of Biology, Drexel University, Philadelphia, Pennsylvania, USA; 5 Department of Internal Medicine, University of Nebraska Medical Center, Omaha, Nebraska, USA; 6 Glenn Biggs Institute for Alzheimer’s & Neurodegenerative Diseases, UTHSCSA, San Antonio, Texas, USA

**Keywords:** hspb1, skn-1, Collagen, *Caenorhabditis elegans*, Naked mole-rat

## Abstract

To explore the role of the small heat shock protein beta 1 (HspB1, also known as Hsp25 in rodents and Hsp27 in humans) in longevity, we created a *Caenorhabiditis elegans* model with a high level of ubiquitous expression of the naked mole-rat HspB1 protein. The worms showed increased life span under multiple conditions and also increased resistance to heat stress. RNAi experiments suggest that HspB1-induced life extension is dependent on the transcription factors *skn-1* (Nrf2) and *hsf-1* (Hsf1). RNAseq from HspB1 worms showed an enrichment in several *skn-1* target genes, including collagen proteins and lysosomal genes. Expression of HspB1 also improved functional outcomes regulated by SKN-1, specifically oxidative stress resistance and pharyngeal integrity. This work is the first to link a small heat shock protein with collagen function, suggesting a novel role for HspB1 as a hub between canonical heat response signaling and SKN-1 transcription.

Protein homeostasis involves multiple processes regulating protein synthesis, folding, and degradation. As an organism ages, there is a decrease in expression of multiple components as well as the overall function of this protein homeostasis network (1). This includes a decrease in molecular chaperones, which aid in protein-folding and preventing formation of deleterious protein aggregates (1). Failure of the protein homeostasis network occurs during aging and many neurodegenerative diseases. A key component of this network, small heat shock proteins (sHSP’s) are molecular chaperones that function not only in protein folding, but also to improve the degradation activity of the proteasome and autolysosome, thereby decreasing disease-associated aggregation.

Comparative biology of aging uses the longest life span reported for an individual of a species (maximum life span, or MLS) as a marker of a species’ life-span potential. The order Rodentia is useful for such studies as it contains a number of well-studied species with a range of MLS across nearly an order of magnitude: The common house mouse (*Mus musculus*) has an MLS of 4 years (2) while the naked mole-rat (*Heterocephalus glaber*) has an MLS of 31 years (3). To better understand the role of molecular chaperones in the age-related decline of protein homeostasis, our lab, in collaboration with the Buffenstein group, previously measured the protein levels of key molecular chaperones in the muscle and liver of various rodent species (4). Heat shock protein beta-1 (HspB1, also known as Hsp25 in rodents and Hsp27 in humans) had the best-fit linear regression between maximum life span and protein expression levels in both tissues. Additionally, HspB1 expression was 2- to 10-fold higher in naked mole-rats compared to mice in other tissues examined, including the brain, lung, and kidney (4,5).

Independent work also examined mechanisms of proteostasis by measuring both the basal and heat-shock-induced expression of HSPs (including HspB1, in cultured fibroblasts from long- versus short-lived species in different mammalian orders. In rodents and marsupials, both basal and heat-shock-induced HSP expression were found to be higher in the longer-lived species, specifically, naked mole-rats versus mice and sugar gliders (*Petaurus brevicaudus*, MLS 18 years) versus opossums (*Monodelphis domestica*, MLS 4.75 years) (6).

Heat shock proteins (HSPs) are molecular chaperones that were first characterized by their dramatic upregulation in response to heat stress (7), but they have since been found to be integral in responding to multiple forms of cellular stress. The suite of HSPs induced depends on both the type of stress (heat, oxidative, UV, etc.) as well as the cellular location wherein the stress occurs (8,9). Small HSPs (12-40 kDa) are characterized by an alpha crystallin domain and an N-terminal RLFDQxFG sequence that enhances oligamerization and generally form large assemblies with other small HSPs (10,11). In mammalian cells, HspB1 protects against oxidative stress (12,13), dependent in part on its ability to form aggregates (14,15).

HspB1 is well-conserved among chordates (16) but there are potentially significant differences in invertebrate orthologs, including the absence of the serine phosphorylation domain in *Caenorhabditis elegans* HSP-25 (Supplementary Data 1). Although HspB1 is expressed at high levels in longer-lived animals (4), no studies have fully examined the effect of high constitutive levels of HspB1. Thus, we created a transgenic strain *C elegans* which would have high ubiquitous expression of the naked mole-rat (NMR) HspB1 protein in order to study the effects of HspB1 expression on life span. This was similar to the engineering approach taken by Sagi and Kim in their study, where they expressed longevity-associated human and zebrafish genes in *C elegans* to extend life span (17). We found that NMR HspB1 expression increased life span as well as resistance to heat and oxidative stresses. RNA sequencing and subsequent epistasis studies suggested that these effects were dependent on SKN-1.

## Results

### Transgenic Expression of HspB1 Increases Life Span and Heat Resistance

**Figure 1.**
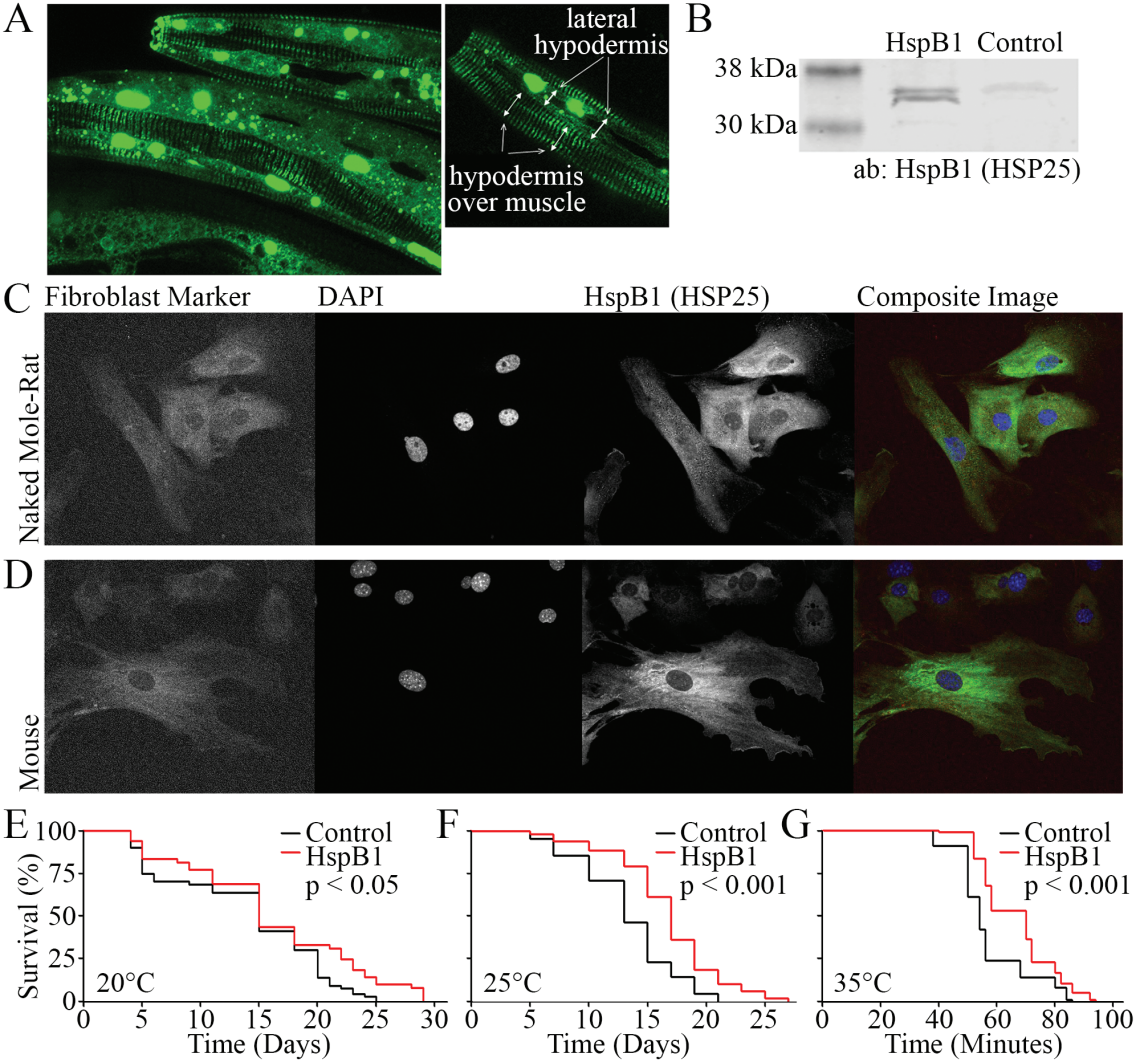
Expression of naked mole-rat HspB1 in *C. elegans* improves life span and health span. (**A**) HspB1-GFP expression is driven by the ubiquitous promoter, sur-5, and drives expression that is both diffuse and punctate. (**B**) HspB1 expression increases protein levels. (**C**) Endogenous HspB1 expression in naked mole-rat fibroblasts. (**D**) Endogenous HspB1 expression in mouse fibroblasts. (**E**) HspB1 expressing animals live longer than control animals when grown at 20°C (*p* < .05) and (**F**) 25°C (*p* < .001). (**G**) HspB1 animals survive longer at 35°C in a heat resistance assay (*p* < .001). Number of animals used in the analyses is shown in parentheses.

To examine the interactions of NMR HspB1 independent of its endogenous environment and its role in health and longevity, we created transgenic *C elegans* that express the naked mole-rat *HspB1* (NMR HspB1) gene fused to GFP under the control of the ubiquitously expressed *C elegans sur-5* promoter. The fusion protein could be seen in multiple tissues, including the hypodermis (Figure 1A), and exhibited diffuse expression along with puncta of varying size. We further confirmed expression of NMR HspB1 protein with Western blotting and observed an enrichment in HspB1::GFP in the transgenic animals compared to vector controls (Figure 1B). Initial assays had suggested that HspB1 tended to form more puncta in naked mole-rat cells than it did in mouse, but we observed relatively diffuse HspB1 distribution in both NMR and mouse fibroblasts (Figure 1C and D and Supplementary Figures 1–3).

NMR HspB1 expression increased life span at both 20°C (*p* < .05) and 25°C (*p* < .01; Figure 1E and F). Median life span was reproducibly increased by at least 25% at 25°C (from an average of 11 days to 15). In addition, NMR HspB1 expression improved heat stress resistance: HspB1 animals placed at 35°C on Day 1 of adulthood showed prolonged survival (25% increase in median, from 9.3 hours to 11.7) compared to control animals (*p* < .001; Figure 1G). Life tables of independent replicates and full statistics are shown in Supplementary Data 2.

### HspB1 Alters Expression of Collagen and Lysosomal Genes

**Figure 2.**
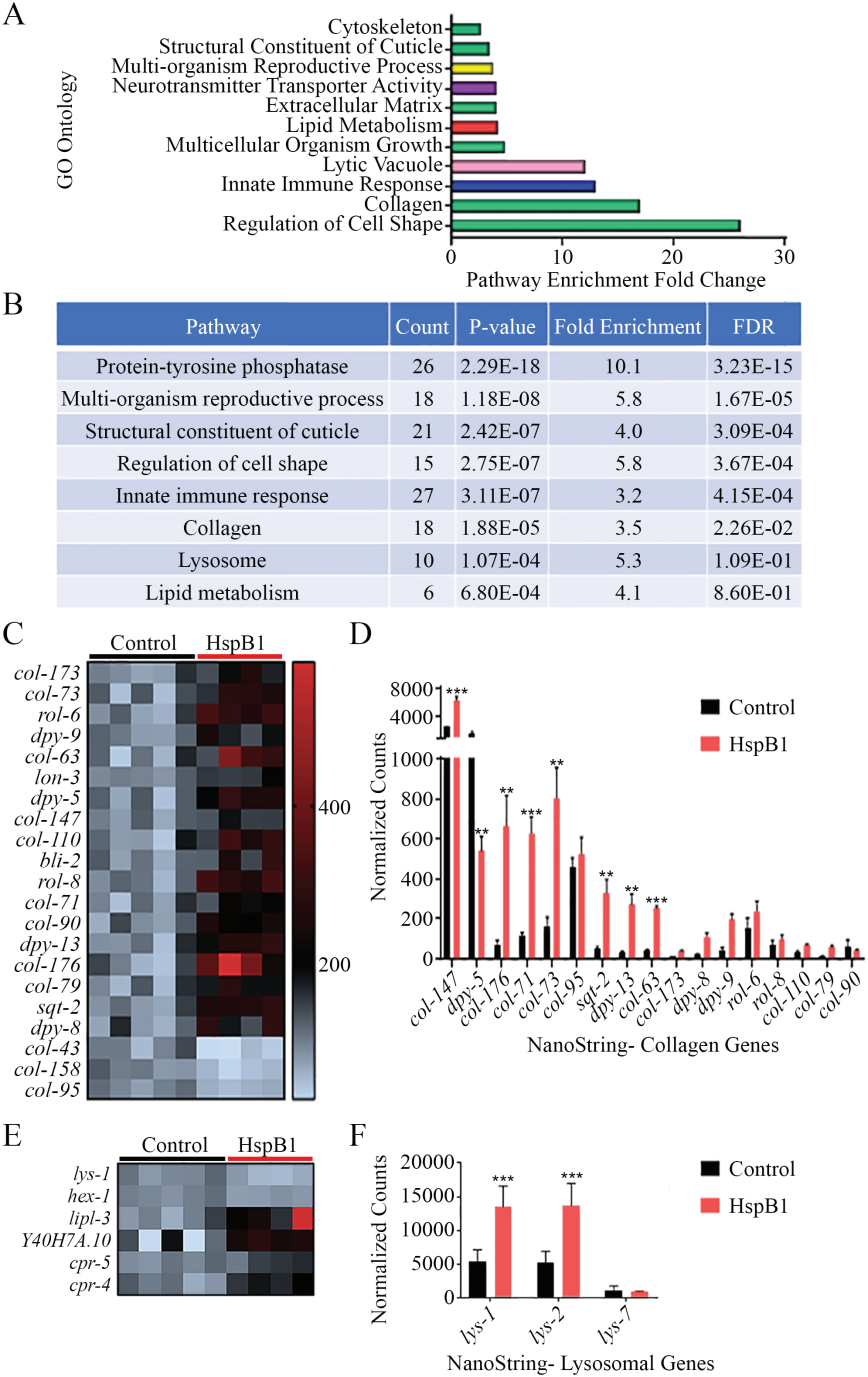
Whole transcriptome sequencing of the HspB1 expressing animals compared to controls. (**A**) Go Ontology enrichment analysis from DAVID shows several altered pathways with an enrichment fold change of >1.5. Shown in green are pathways associated with collagen genes. (**B**) Significantly altered pathways are listed with the count of genes in the given pathway, the *p*-value, the fold enrichment, and the false discovery rate (FDR). (**C**) The heat map shows the differentially expressed collagen genes that were statistically significant (*p* adjusted <.05). Genes that increased in the HspB1 expressing animals are in red and decreased in blue. The intensity shows the ratio of the expression change, with the control expression set to 100%. A similar heat map is shown for (**E**) lysosomal genes as well. (**D**) RNAseq collagen hits and (**F**) lysosomal hits were validated with NanoString nCounter technology. The red bars show the average normalized counts for 5 biological replicates of HspB1-GFP populations while the black bars show the controls. (*p < .05, **p < .01, ***p < .001).

To examine the mechanisms of life extension and increased heat resistance, we sequenced the whole transcriptome of our NMR HspB1 and vector control animals at Day 1 of adulthood. We identified 588 differentially expressed genes, with an adjusted *p*-value <.05 (Figure 2, Supplementary Figure 4, and Supplementary Data 3). Using the DAVID software, we categorized the differentially expressed genes into several enriched pathways with a false discovery rate of <0.01, a fold enrichment >1.5, and a *p*-value <.01. Gene Ontology analysis revealed that genes involved in collagen proteins, the cuticle, and lysosomal function were all differentially expressed (Figure 2A and B).

In general, collagen genes showed increased expression in the NMR HspB1 animals compared to controls (Figure 2C). We confirmed upregulation of *col-147*, *col-176*, and *col-71* using NanoString nCounter technology (Figure 2D). In addition, lysosomal gene expression was altered (Figure 2E), although the direction was more varied in this group of genes. Two of the lysosomal genes that were enriched in our RNAseq data set (*lys-1* and *lys-2*) were also validated with NanoString nCounter analysis (Figure 2F).

We noted that many of the upregulated genes are targets of the transcription factor SKN-1 (Nrf2 in mammals). Specifically, SKN-1 has been shown to regulate expression of collagen, immunity, and lysosomal function genes (18–20).

### HspB1 Life-span Extension May be Partly Dependent on *skn-1*, *hsf-1*, and *daf-16*

**Figure 3.**
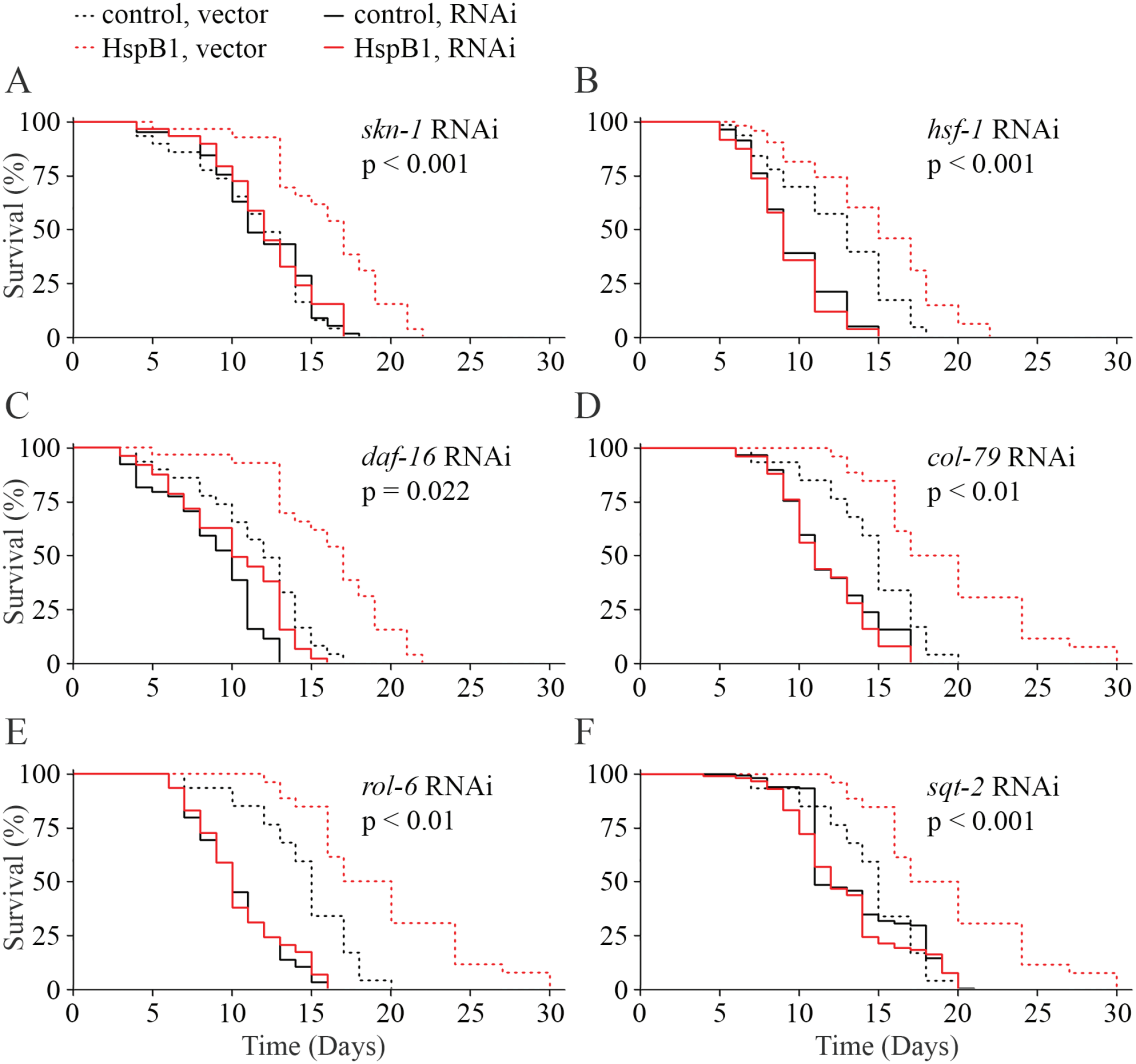
HspB1 life-span extension is dependent on skn-1 and collagen genes. (**A**) HspB1 life extension is abolished on skn-1 RNAi (**A**) and hsf-1 RNAi (**B**) but only attenuated on daf-16 RNAi (**C**). Collagen gene col-79 (**D**), rol-6 (**E**), and sqt-2 (**F**) RNAi also prevents HspB1 life extension. All *p*-values shown are Cox Hazard test for strain:treatment interaction. Additional replicates and statistics are included in Supplementary Data 2.

Based on our RNAseq results that suggested SKN-1 pathways were altered in the NMR HspB1 worms, we wanted to test the dependency of life extension on SKN-1. We started gene knockdown via feeding RNAi only when the animals had reached the L4 larval stage to minimize effects on development. Under these conditions, *skn-1* RNAi had no effect on control life span while HspB1 induced life extension was completely abolished (*p* < .0001; Figure 3A).

Heat shock factor 1 (HSF-1) is the well-conserved canonical regulator of heat shock proteins and contributes to life-span determination (21). On *hsf-1* RNAi, both HspB1 and control animals showed dramatically shortened life span (*p* < .0001; Figure 3B).

DAF-16 (FOXO) is another transcription factor shown to be critical life extension in numerous models, often in conjunction with SKN-1 or HSF-1 (22,23). While *daf-16* RNAi attenuated most of the life extension induced by HspB1 (*p* < .0001), the HspB1 animals still lived significantly longer than control on *daf-16* RNAi (*p* < .01; Figure 3C).

In order to test whether knocking down any of these 3 transcription factors—*skn-1*, *hsf-1* and *daf-16*—by RNAi affected sur-5p::NMR.HspB1::GFP expression, we imaged Day -1-adult worms that had, like those in our life-span assays, been transferred to each RNAi treatment at L4. Surprisingly, *skn-1* RNAi reduced HspB1::GFP abundance by a third while neither *hsf-1* nor *daf-16* RNAi had any significant effect (Supplementary Data 5).

We moved on to testing the requirements for genes downstream in the life extension induced by HspB1. Our RNAseq analysis showed that collagen genes were upregulated in the HspB1 animals compared to controls. We confirmed the importance of the top collagen genes by testing the life span of the HspB1 versus control animals on RNAi for 3 upregulated collagen genes: *col-79*, *rol-6*, and *sqt-2*. All 3 collagen RNAi’s shortened the life span of HspB1 worms significantly more than control (*p* < .01; Figure 3D–F; See Supplementary Data 2 for full statistics).

### HspB1 Expression Improves the Function of SKN-1-associated Phenotypes

**Figure 4.**
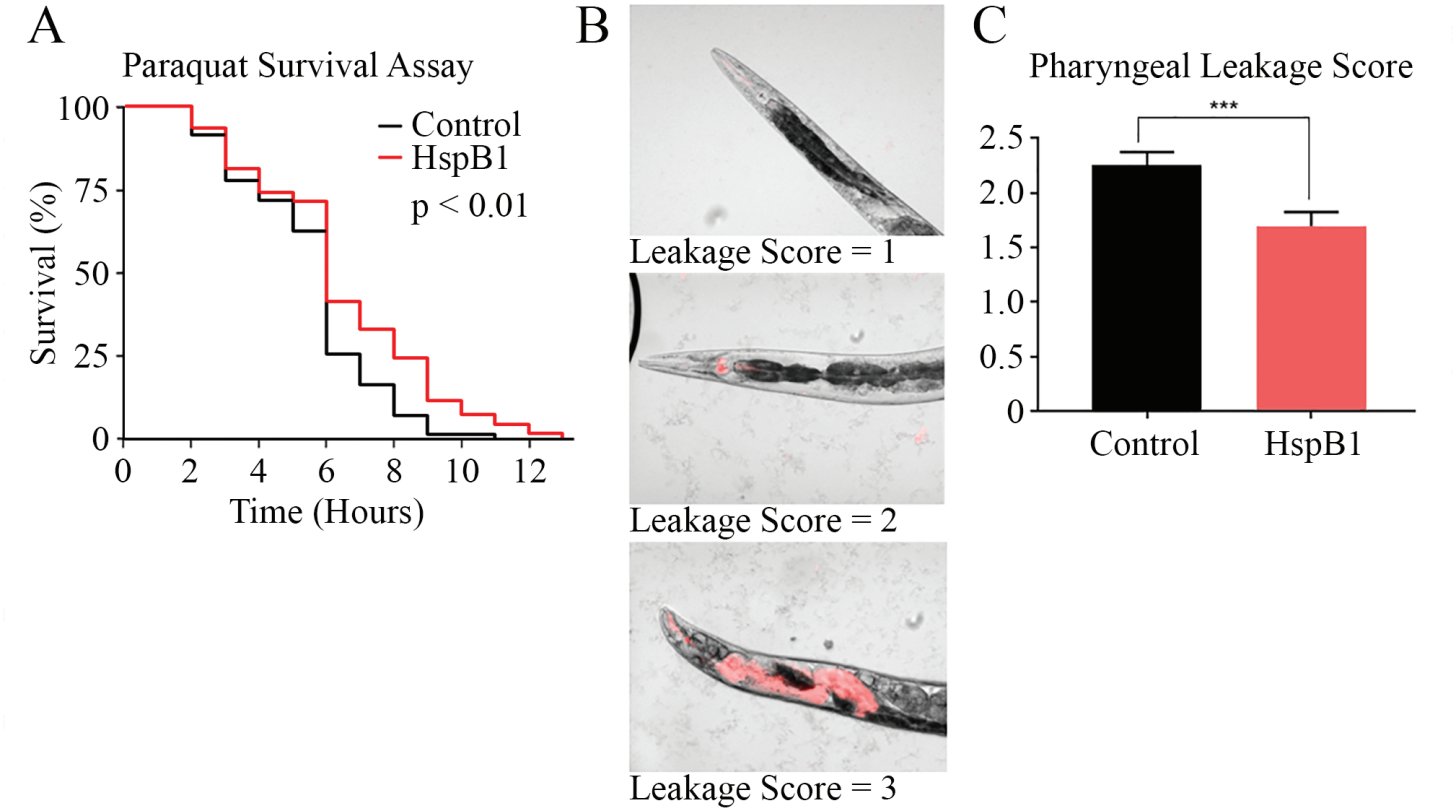
HspB1 expressing worms have improved SKN-1 associated functions. (**A**) HspB1 expressing animals have increased survival on 10 mM paraquat (*p* < .01). (**B**) HspB1 worms also have a decrease in pharyngeal leakage. Leakage scores were assigned with a one given to no visible infiltration (top, HspB1 animal, Day 10 of adulthood), a two if there were RFP-positive pharyngeal inclusions (middle, control animal, Day 10 of adulthood) and a score of three if there was full leakage (bottom, control animal, Day 10 of adulthood). (C) The average leakage score for Day 10 of adulthood was lower in HspB1 animals compared to controls (*p* < .001), with an *n* of 10 per group, 3 biological replicates plotted. Number of animals used in the analyses is shown in parentheses.

Since the collagen genes upregulated by NMR.HspB1 and required for resultant life extension are reported to be regulated by SKN-1 (18–20), we next tested whether HspB1 overexpression also increases SKN-1-related functions. In addition to the pathways enriched in our RNAseq analysis, SKN-1 is responsible for activating responses to oxidative stress (20). To test whether our HspB1 worms were also resistant to oxidative stress, we assayed survival on paraquat and found that HspB1 animals survived marginally longer than control animals (*p* < .05; Figure 4A).

Based on the increase in collagen genes seen in our RNAseq and the effect of collagen RNAi’s on the HspB1 animals’ life span, we next tested collagen function by assaying structural integrity of the pharynx over time. We used *Escherichia coli* that express red fluorescent protein to visualize the leakage of bacteria into the pharynx (24). Animals were aged to Day 10 of adulthood, and then imaged and categorized. A leakage score of one represents no pharyngeal leakage (Figure 4B, top); a leakage score of 2 shows animals with fluorescent inclusions building in the pharynx (Figure 4B, middle); a leakage score of 3 shows complete pharyngeal leakage (Figure 4B, bottom). HspB1 animals had significantly less pharyngeal leakage than control animals (*p* < .001; Figure 4C).

## Discussion

Previously, the Buffenstein group showed that HspB1 is correlated with maximum life span in rodents and is highly expressed in the long-lived naked mole-rat, although the mechanism by which it relates to life span is not well understood (4). We show here that even though HspB1 frequently acts in concert with other small heat shock proteins, overexpression of the naked mole-rat HspB1 alone is enough to improve life span and both heat and oxidative stress response in an invertebrate model.

The NMR HspB1 transgenic animal has ubiquitous expression of HspB1::GFP, which could be seen both diffusely and in discrete puncta. Further research is needed to determine whether puncta formation is a feature of NMR HspB1 or an artifact of ubiquitous high expression. Whether endogenous HspB1 behaves differently in naked mole-rats compared to mice, dependent perhaps on cell-type or stressor, is an area of ongoing investigation. In the *C elegans* hypodermis, HspB1::GFP appeared to localize to the fibrous organelles (Figure 1A), which anchor fibrils that extend from the basal lamina of the cuticle to muscles via the M-line and dense bodies of the sarcomere (25,26). Stabilization of the hypodermis may contribute to the improved longevity and stress resistance we observed. The HspB1 animals live longer at both 20°C and 25°C, with greater life extension at 25°C, a condition of low-grade chronic heat stress. Additionally, the HspB1 worms lived longer in extreme heat stress of 35°C, highlighting the importance of HspB1 in the heat stress response.

Further studies are needed to determine whether benefit is derived from increased expression of HspB1 or specifically from expression of naked mole-rat HspB1. *Caenorhabiditis elegans* does have a small heat shock protein orthologous to HspB1; however, there are significant differences in the sequence, including the absence of the serine phosphorylation site that is conserved among chordates (Supplementary Data 1). Uniquely, the naked mole-rat HspB1 has an alanine repeat in the region just before the conserved αβ crystallin domain (Supplementary Data 1). It is possible that this repeat enhances NMR HspB1’s ability to form oligomers, resulting in the puncta seen in both the NMR primary cells and transgenic worm line. Enhanced oligomeric activity could play a role in forming the protective aggresomes that have been observed to protect the cells of long-lived species from toxic aggregation-prone proteins (27). Whether NMR HspB1 confers special protection beyond other species’ and, if so, what features contribute to this, is an area of ongoing research in our lab.

### HspB1 and Altered Gene Expression

NMR HspB1 expression altered multiple gene pathways, including collagen and lysosomal genes. RNAi life-span assays provided further evidence for the involvement collagen genes in NMR HspB1-induced life extension. Significantly, we also observed improved oxidative stress resistance to paraquat and enhanced cuticle stability with reduced pharyngeal leakage at middle age (Day 10 of adulthood).

The results of our tests for the requirement of specific transcription factors in NMR HspB1-induced life extension were inconclusive. RNAi knockdown of *skn-1* reduced the life span of both HspB1 and control worms, with far greater effect in HspB1. However, this may be largely or even completely due to *skn-1* RNAi reducing the abundance of sur-5p::NMR.HspB1::GFP. This effect was unexpected since *skn-1* RNAi has been reported to have no effect on sur-5::GFP even when the worms are treated from an L1 stage (28); we did not expect the abundance of *skn-1* to alter transcription from the *sur-5* promoter.

In contrast to *skn-1*, *hsf-1* RNAi dramatically shortened life span in both lines and had no effect on HspB1::GFP abundance. This reduced life span was expected as *hsf-1* RNAi causes a rapid-aging phenotype, including degradation of the pharynx and early mortality (29). However, while *hsf-1* RNAi always reduces life span, it does not always completely abrogate life extension as we observe here with our HspB1 worms: the long-lived mutants *eat-2(ad1116)* (a dietary restriction model) and *isp-1(qm150)* (inhibited mitochondrial respiration) still survive longer than wild-type worms on *hsf-1* RNAi, while the otherwise long-lived *daf-2(e1370)* (inhibited insulin signaling) mutant, like our HspB1 line, is reduced to a life span indistinguishable from wild-type (22).

On *daf-16* RNAi, the life-span reduction in HspB1 was greater than in control worms and Cox Hazard analysis confirmed a significant strain:treatment interaction (p = 0.022). This could suggest that HspB1 induced life extension is only partially dependent on DAF-16. However, the RNAseq data have not been revelatory of any such role. Most genes that have been reported upregulated dependent on DAF-16 (30), including the canonical targets *mtl-1* and *sod-3* (31,32), were not upregulated in HspB1 worms compared with controls (Supplementary Data 4). Note that while we did not observe a significant effect of *daf-16* RNAi on sur-5p::NMR.HspB1::GFP when gene knockdown was started at L4 stage, it has been reported that *daf-16* RNAi from L1 stage reduces sur-5::GFP expression by 10% (28).

### Collagens and Cuticle in Life Span

Other groups have shown that collagen genes are SKN-1-dependent and that collagen remodeling contributes to longevity (19). Several longevity-promoting interventions, such as *daf-2* mutations or RNAi, increase collagen gene expression in a SKN-1-dependent manner, and that collagen overexpression increases life span (19). Cuticle leakage is a major contributor to *C elegans* mortality (24). Therefore, interventions that strengthen the cuticle should increase life span by preventing or delaying the onset of cuticle dysfunction. Our data support this model.

Dodd et al. showed that RNAi disruption of specific bands of collagen co-activated the detoxification, hyperosmotic, and antimicrobial response genes. Both detoxification and the hyperosmotic stress response required *skn-1* (18). Furthermore, HspB1-induced life extension is dependent on SKN-1’s collagen gene transcriptional targets. Our work lends support to a SKN-1-dependent role of collagens and the cuticle in life extension, with HspB1 expression serving as a catalyst for this pathway.

### HspB1 and Human Disorders

Aging and many age-related neurodegenerative disorders are characterized by protein aggregation and general dysfunction in the protein homeostasis network (1). Overexpression of the human HspB1 helps decrease protein aggregation in mouse models of Huntington’s disease, although the mechanism is not well understood (33). It is unclear if HspB1 acts directly by interacting with aggregates that form or if HspB1 acts indirectly through other genes such as SKN-1 to ameliorate the negative effects of protein aggregation in neurodegeneration. Recent in vitro analysis by 2 groups has shown that mouse HspB1 (HSP25) can bind to different species of tau and regulate its aggregation (34,35), but not to deleterious tau proteins already in aggregates. Further work is needed to explore the role of HspB1 in age-associated disease states in vivo and the composition of the HspB1 aggresome.

## Experimental Procedures

### Cell Culture and Imaging

One-day-old naked mole-rat and mouse pups were rinsed with Wescodyne solution and with 70% ethanol prior to decapitation. A 1–2 mm piece of dorsal skin was removed with dissecting scissors and then rinsed with 1× tissue culture grade PBS containing 1× primocin (Invitrogen). The skin biopsy was then rinsed in 70% ethanol, followed twice more in 1× tissue culture grade PBS with 1× primocin. The tissue was then finely minced with a sterile razor in 0.25 mL 0.25% trypsin/EDTA. Another 0.25 mL 0.25% trypsin was added to the dish before incubating for 15 minutes in 5% CO_2_ at 35°C (naked mole-rat) or 37°C (mouse).

After incubation, trypsin was neutralized with 10% FBS DMEM + 1× primocin. The media was then carefully aspirated out of the dish without disturbing the larger tissue pieces. One to 2 drops of 10% FBS DMEM + 1× primocin was then added to the explant and returned to the respective tissue culture incubator for 7 to 10 days to allow fibroblasts to grow out of the explant. At this point, the cells were rinsed with 1× HBSS, trypsinized, rinsed from the dish with 10% FBS DMEM + 1× primocin, and then centrifuged to obtain a pellet. The cells were then resuspended in fresh 4 mL of 10% FBS DMEM + 1× primocin and plated into a T25 flask which is labeled as passage 1.

Cells were incubated with primary antibodies overnight at 4°C prior to imaging with 25× objective. The Fibroblast marker employed was ER-TR7 (Santa Cruz sc73355 lot#J3019 at 1:100 in 5% normal goat serum PBS; 561 laser: 300; PMT 700. To measure HSP25, we used Enzo AD1-SPA-801-F lot#07031822 at 1:50 in 5% normal goat serum PBS; 488 laser: 200; PMT 700.

DAPI at 1uM final for 5 minutes post-stain; 405 laser: 300; PMT 700. Secondary antibodies used were Alexa mouse 488, Alexa rat 568, and Alexa rabbit 488 all used at 1:200 final dilution.

### Worm Maintenance Conditions

Worms were maintained using standard techniques (36) and experiments were performed at 20°C unless otherwise indicated. The following strains were used in this study: wild-type (N2); HT1593 (*unc-119(ed3)*), which were used to create transgenic lines; KAR1 (*satIs1*[*sur-5p*::HspB1::GFP]), which express naked mole rat HspB1 fused to GFP under the ubiquitous *sur-5* promoter; and KAR2 (*satIs2*[*sur-5p*]), which serve as the vector controls.

### Generation of Transgenic Animals

A synthetic cDNA encoding the HspB1 protein from the NMR was designed with *C elegans* optimized codon usage. This cDNA was then purchased from IDT (Coralville, IA) as a synthetic gene cloned into the pIDTSmart-amp vector. The cDNA was removed from the pIDT vector using Acc65I and subcloned into the pPD158.87 vector (Addgene Inc., Cambridge, MA), which contains the *C elegans* ubiquitous *sur-5* promoter, to generate a *sur-5p::HspB1::GFP* transgene. The *unc*-*119* gene from *C. briggsae* was then substituted for the ampicillin resistance gene on the vector backbone via homologous recombination using p*unc*-*119cbr* vector as previously described to provide a selectable marker (37). The resulting plasmid was then used to bombard HT1593 (*unc*-*119*(*ed3*)) as described (38). Transgenic strains were identified by rescue of the *unc*-*119* mutant phenotype, and led to the establishment of the strain KAR1 (*satIs1*[*sur-5p*::HspB1::GFP] that is used in this study. We also generated a negative control transgene which contained the HspB1 cDNA in reverse orientation in the same vector. This vector was then used to generate the negative control strain KAR2 (*satIs2*[*sur-5p*]), by bombardment as described above. Both strains were outcrossed with N2 at least 4 times prior to use.

### Life-span Assay

Animals were synchronized with hypochloride treatment and placed onto NGM plates seeded with OP50. For RNAi experiments, plates were instead seeded with HT115 and at the L4 stages, worms were transferred to plates which additionally contained 50 μM of 5-fluorodeoxyuridine (FUDR) to prevent reproduction. Worms were transferred to new plates every other day for the first 6 days of adulthood. Missing animals and those with internally hatched larvae were removed from the analysis. Animal survival was scored every day by response to touch and was marked as dead if they did not respond. There were at least 25 animals per group, with at least 3 biological replicates per experiment. We used R to generate all graphs and perform log-rank testing for comparing survival curves Cox Hazard analysis to test for significant strain:treatment interaction when comparing HspB1 transgenic worms to controls on RNAi.

### Heat Resistance Assay

Heat resistance assays were performed on OP50 seeded NGM plates placed at 35°C. Animal survival was scored every hour by the response to touch and was marked as dead if they did not respond. We used Prism6 (Graphpad Software, La Jolla, CA) to generate all graphs and perform log-rank testing for comparing survival curves.

### Fluorescence Microscopy

Animals were mounted on 2% agar pads, immobilized with sodium azide, and imaged with Zeiss LSM700 microscope, using 1.4NA 63× oil objective. 12-bit confocal z-stacks were reconstructed in ImageJ as 3D projections.

### *C elegans* Lysis

Approximately 800 synchronized worms were used for each condition. Eggs were hatched on NGM plates and animals were aged to Day 0 of adulthood at 20°C. Worms were then washed from the plate with S-BASAL, gently pelleted with centrifugation at 3 000 rpm for 1 minute, and washed again in S-BASAL to ensure removal of any bacteria. Worms were then resuspended in 250uL of resuspension buffer (10 mM HEPES, 10 mM NaCl, 1.5 mM MgCl2, 2 mM ATP, 0.5M DTT, pH 6.2), with 5% glycerol.

After resuspension, animals were lysed with a Tissue Lyser. For the Tissue Lyser, 3 or 5 mm beads were used at a speed of 50 Hz for 1 minute. Following the initial homogenization, worms were placed at 4°C for 30 minutes on a vigorous shake. Then, the mechanical homogenization was repeated. The insoluble fraction was then pelleted by centrifugation at 5 000 rpm for 5 minutes, and the supernatant was collected. Protein concentration was determined using the standard BCA assay (Pierce, Waltham, MA).

### Protein Extraction and Western Blotting

Standard Western blotting methods were used. Twenty micrograms of total worm lysate from disruption of 800–1 000 whole worms described above were boiled in Laemmli buffer and loaded onto a gradient SDS-PAGE gel, separated, and transferred onto PVDF membrane. Membranes were stained with LI-COR Total Protein Stain and imaged using the LI-COR Odyssey. Then, membranes were probed with anti-HspB1 antibody (ADI-SPA-801, ENZO Life Sciences, Farmingdale, NY;1:1 000 overnight at 4°C), followed by a fluorescent secondary antibody and imaging on the LI-COR Odyssey and analysis on LI-COR Imaging software.

### RNA Extraction and Whole Transcriptome RNA Sequencing

Five biologically independent populations of KAR1 and KAR2 worms were used. Worms were synchronized with hypochloride treatment and grown on standard NGM plates at 20°C. At Day 1 of adulthood, worms were washed from plates with S-BASAL and resuspended in Trizol. Total RNA was extracted using Direct-zol RNA Mini Prep Plus kit and quantified. RNA was sent to Genewiz for sequencing.

Using Galaxy, sequence files were aligned to the *C elegans* genome with HISAT and the overlap of the reads with specific genes were counted with HTSEQ-COUNT. With DESeq2, differential expression based on a model using the negative binomial distribution was calculated, and we calculated the normalized counts for each sample. Genes with an adjusted *p*-value of <.05 were considered significantly differentially expressed. Pathway analysis was performed with DAVID to identify enriched gene clusters and biological pathways (39).

### NanoString Assay

Gene expression was measured as previously described (39). NanoString Technologies (Seattle, WA) synthesized code sets that recognize target genes. The nCounter system was used to assess the amount of transcript for each gene in the panel present in 100 ng of total RNA. The analysis was done with the NanoString nSolver system, with normalization to the geometric mean of housekeeping genes (*act-1, ama-1, nhr-23,* and *pmp-3*), and significance was tested with 1-way ANOVA analysis with the nSolver software. RNA was isolated from total worms at Day 1 of adulthood as described above. Five biological replicates with approximately 1 000 animals per group were used.

### RNAi Treatment

The effect of RNAi on life span was measured as previously described (39). All RNAi clones were retrieved from the Ahringer RNAi library and confirmed with sequencing. RNAi bacterial cultures grown overnight were spotted onto NGM plates with 50 μg/mL carbenicillin, 1 mM IPTG, and ±50 μM of 5-fluorodeoxyuridine (FUDR) as indicated. Approximately 40 L4 animals were placed onto the spotted plates and placed at 20°C. We then assayed life span as described above. Prism6 (Graphpad Software) was used to generate all graphs and perform log-rank testing for comparing survival curves.

### Oxidative Stress Assay

Oxidative stress was assessed as previously described (40). Age synchronized Day 1 adult worms were washed off NGM plates with S-BASAL. Four groups of approximately 20 animals each were suspended in 500 μL of 100 mM paraquat in S-BASAL, placed at room temperature, and then monitored each hour. Survival was determined as the ability to thrash in liquid culture. We used Prism6 (Graphpad Software, La Jolla, CA) to generate graphs and perform log-rank testing to compare survival curves.

### Pharyngeal Leakage Assay

*Escherichia coli* OP50 expressing red fluorescent protein (OP50-RFP) was kindly provided by Dr. Yuan Zhao and the pharyngeal leakage assay run as previously reported (24). In summary, we cultured OP50-RFP was in the presence of 25 μg/mL tetracycline and then resuspended the bacteria in LB broth without antibiotics before seeding NGM plates. Worms were raised from eggs on this OP50-RFP and transferred to fresh plates every 2–3 days. At Day 10 of adulthood, worms were mounted on 2% agar pads with 10 mM sodium azide and imaged, as described above. The amount of pharyngeal leakage was scored, with a score of 1 for worms with no RFP leakage, a score of 2 for RFP-positive inclusions, and a score of 3 for full RFP leakage into the pharynx. We used Prism6 (Graphpad Software, La Jolla, CA) to compare the 2 groups with a Student’s *t*-test.

## Supplementary Material

glab296_suppl_Supplementary_Data_1

glab296_suppl_Supplementary_Data_2

glab296_suppl_Supplementary_Data_3

glab296_suppl_Supplementary_Data_4

glab296_suppl_Supplementary_Data_5

glab296_suppl_Supplementary_Figure_1

glab296_suppl_Supplementary_Figure_2

glab296_suppl_Supplementary_Figure_3

glab296_suppl_Supplementary_Figure_4

glab296_suppl_Supplementary_Figure_5
