# Supplementary material for: HspB1 Overexpression Improves Life Span and Stress Resistance in an Invertebrate Model
Source: J Gerontol A Biol Sci Med Sci. 2021 Oct 5;77(2):268–75. doi: 10.1093/gerona/glab296 (PMC8824566; doi:10.1093/gerona/glab296)
Supplement: glab296_suppl_Supplementary_Data_1 [file glab296_suppl_supplementary_data_1.docx]

HspB1 sequence homology among chordates

The αβ crystallin domain and conserved site of serine phosphorylation are highlighted.

Homo sapiens (human) CAG38728.1

Heterocephalus glaber (naked mole-rat) XP_004840151.1

Mus musculus (mouse) AAH99463.1

Myotis lucifugus (little brown bat) XP_014310876.1

Monodelphis domestica (opossum) XP_001378972

Columba livia (rock dove) PKK23707.1

Gallus gallus (chicken) NP_990621.1

Xenopus tropicalis (western clawed frog) NP_001072817.1

Danio rerio (zebrafish) AAI64999.1

CAG38728.1 mterrvpfsllrgpswdpfrdwyp-hsrlfdqafglprlpeewsqwlggsswpgyvrplp 59

XP_004840151.1 mterrvpfsllrspswdpfrdwypshsrifdqafglprlpeewsqwfg--gwpgyvrslp 58

AAH99463.1 mterrvpfsllrspswepfrdwypahsrlfdqafgvprlpdewsqwfsaagwpgyvrplp 60

XP_014310876.1 mterrvpfsllrspswdpfrdwypvhhrlfeqafgmprlpeewsqcfshsgwpgyvrplp 60

XP_001378972 mterrvpftflrspswdpfkdwypvgsrlfdqsfglprlpeewcqwpshtswpgyvrmlp 60

PKK23707.1 maerrvpftflrspswdpfrdwyhg-srlfdqsfgmphipedwykwpsgsswpgyfrllp 59

NP_990621.1 maerrvpftfltspswepfrdwyhg-srlfdqsfgmphipedwykwpsgsawpgyfrllp 59

NP_001072817.1 mserripfsflrtpswdpfrdwyqgtsrlfdqsfgmpripedwyqwps-tswpgyvrmlp 59

AAI64999.1 maerripfsfmrspswdpfrdwyqg-srlfdqsfgmpalseemltfps-thwpgymrpfg 58

*:***:**::: ***:**:*** *:*:*:**:* : :: . ****.* :

CAG38728.1 paaiespa----------vaapaysralsrqlssgvseirhtadrwrvsldvnhfapdel 109

XP_004840151.1 paaaaaaata-------aepapaysralsrqlssgvseirhtpdrwrvsldvnhfapeel 111

AAH99463.1 aataegpaav-------tlaapafsralnrqlssgvseirqtadrwrvsldvnhfapeel 113

XP_014310876.1 aaavegapa---------vaapaysralsrqlssgvseiqhtpdrwrvsldvnhfapeel 111

XP_001378972 splaeqlpaaaaplvppptagqaysralsrqlsrgiseiqhtadrwkvtldvnhfapeel 120

PKK23707.1 resalmp----------------ygqalsrqlssgiseirqtpdswkvtldvnhfapeel 103

NP_990621.1 sesallpa----pgs-------pygrals-elssgiseirqsadswkvtldvnhfapeel 107

NP_001072817.1 sqsmevvp----pttpagatapdfnralsrqlssgiseirqtsdqwkisldvnhfapeel 115

AAI64999.1 hpdfaa--lmqgppvmppmmtpsygralsrqlssgmsevkqtgdswkisldvnhfspeel 116

:.:**. :** *:**:::: * *:::******:*:**

CAG38728.1 tvktkdgvveitgkheerqdehgyi**s**rcftrkytlppgvdptqvssslspegtltveapm 169

XP_004840151.1 tvktkdgvveitgkheerqdehgyi**s**rcftrkytlppgvdptlvvsslspegtltveapi 171

AAH99463.1 tvktkegvveitgkheerqdehgyi**s**rcftrkytlppgvdptlvssslspegtltveapl 173

XP_014310876.1 tvktkegvveisgkheerqdehgyi**s**rcftrkytlppgvdptlvssslspegtltveapm 171

XP_001378972 tvktkdgvveitgkheerqdehgfi**s**rcftrkyslppgvdptlvvsslspdgtlsveapl 180

PKK23707.1 vvktkdniveitxxxeekqdehgfi**s**rcftrkytlppgveatavrsslspdgmltveapl 163

NP_990621.1 vvktkdniveitgkheekqdehgfi**s**rcftrkytlppgveatavrsslspdgmltveapl 167

NP_001072817.1 viktkdgiveitgkheekqdehgfi**s**rcftrkytlppgvdinkvasslspdgiltveapl 175

AAI64999.1 nvktkdgvleitgkheerkdehgfi**s**rcftrkytlppgvdsekissclspegvltveapl 176

:***:.::**: **::****:*********:*****: : *.***:* *:****:

CAG38728.1 pklatqsneitipvtfesraqlggpeaaksdetaak 205

XP_004840151.1 pkv-tqsaeitipvtfearaqiggseagkpeqsgak 206

AAH99463.1 pkavtqsaeitipvtfearaqiggpeagkseqsgak 209

XP_014310876.1 pkpanqsseitipvtfearaqlggpeagkpeqsgaq 207

XP_001378972 pkpaiqsaevtipvtfesraeiggaetkkqgeaaak 216

PKK23707.1 pkpaiqsaeitipvtvesqakepakk---------- 189

NP_990621.1 pkpaiqsseitipvtveakkeepakk---------- 193

NP_001072817.1 pkpaiqsaeiaipitfqsraeigtteakkgeeatkk 211

AAI64999.1 pkpaiqapevnipvnkttvsttk------------- 199

** *: *: **:. :

Caenorhabditis elegans HSP-25, NP_001367469.1 (isoform b), sequence alignment

NP_001367469.1 mserridvnrsnysvid-nefgnmrdrfeqemrrvee--emkrlrsefegyrpnggppaa 57

CAG38728.1 mterrvpf-----sllrgpswdpfrdwyp-hsrlfdqafglprlpeewsq---------- 44

XP_004840151.1 mterrvpf-----sllrspswdpfrdwypshsrifdqafglprlpeewsq---------- 45

AAH99463.1 mterrvpf-----sllrspswepfrdwypahsrlfdqafgvprlpdewsq---------- 45

*:***: . *:: .: :** : . * .:: : ** .*:.

NP_001367469.1 isnqpynaysntsshhetsnrtggfgsplpppsfhgpsdlmahrptydpyldnl-kspli 116

CAG38728.1 ---------------wlggsswpgyvrplppaaiespa---vaapaysralsrqlssgvs 86

XP_004840151.1 ---------------wfg--gwpgyvrslppaaaaaaataaepapaysralsrqlssgvs 88

AAH99463.1 ---------------wfsaagwpgyvrplpaataegpaavtlaapafsralnrqlssgvs 90

*: ** : . : *::. *.. .* :

NP_001367469.1 kdesdgktlrlrfdvanykpeevtvktidnrllvhakheektpqr-tvfreynqefllpr 175

CAG38728.1 eirhtadrwrvsldvnhfapdeltvktkdgvveitgkheerqdehgyi**s**rcftrkytlpp 146

XP_004840151.1 eirhtpdrwrvsldvnhfapeeltvktkdgvveitgkheerqdehgyi**s**rcftrkytlpp 148

AAH99463.1 eirqtadrwrvsldvnhfapeeltvktkegvveitgkheerqdehgyi**s**rcftrkytlpp 150

: . . *: :** :: *:*:**** :. : : .****: :: : * :.::: **

NP_001367469.1 gtnpeqisstlstdgvltveaplpqlaiqq----------------------------- 205

CAG38728.1 gvdptqvssslspegtltveapmpklatqsneitipvtfesraqlggpeaaksdetaak 205

XP_004840151.1 gvdptlvvsslspegtltveapipk-vtqsaeitipvtfearaqiggseagkpeqsgak 206

AAH99463.1 gvdptlvssslspegtltveaplpkavtqsaeitipvtfearaqiggpeagkseqsgak 209

*.:* : *:** :*.******:*: . *.

Sequence alignments were generated with the EMBL-EBI Clustal Omega multiple sequence alignment tool (Madeira et al., 2019).
