# Supplementary figures and images for: HspB1 Overexpression Improves Life Span and Stress Resistance in an Invertebrate Model
Source: J Gerontol A Biol Sci Med Sci. 2021 Oct 5;77(2):268–75. doi: 10.1093/gerona/glab296 (PMC8824566; doi:10.1093/gerona/glab296)

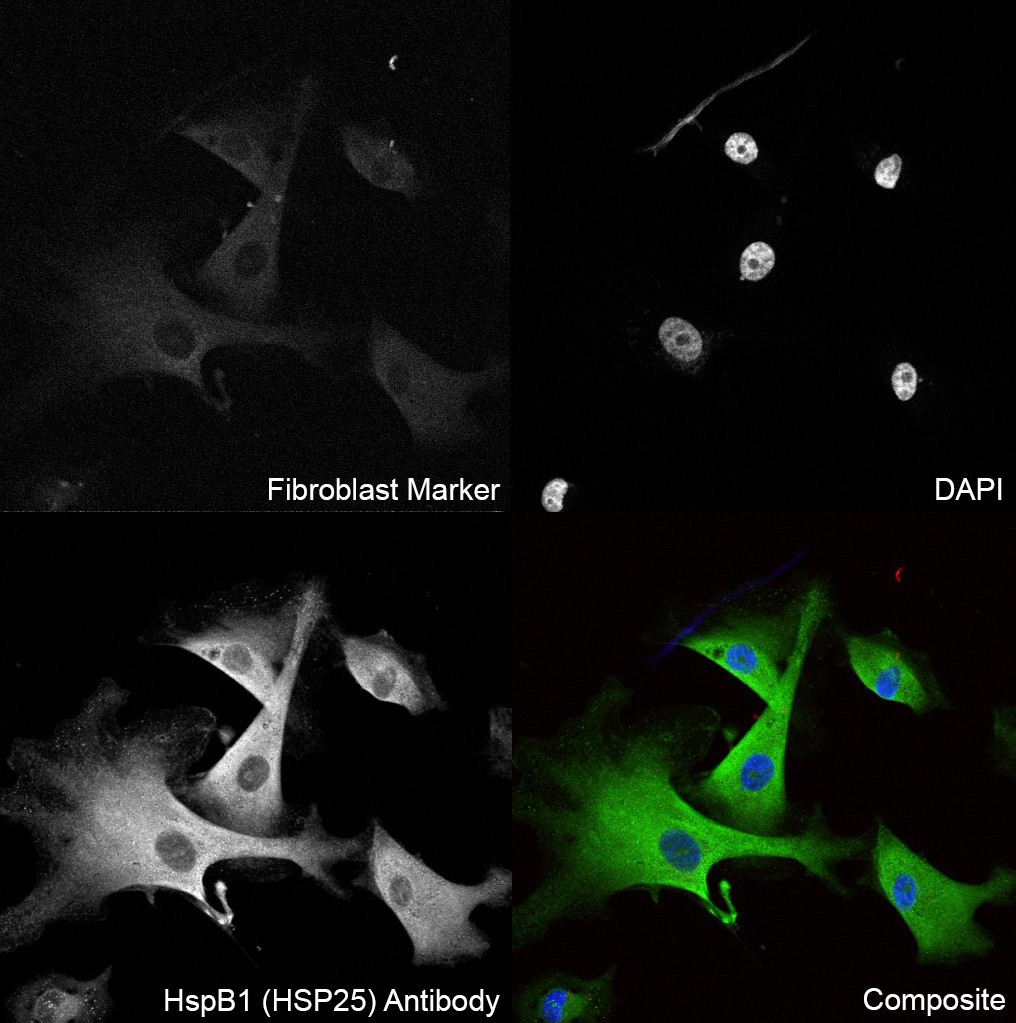

Supplement: glab296_suppl_Supplementary_Figure_1 [file glab296_suppl_supplementary_figure_1.jpeg]

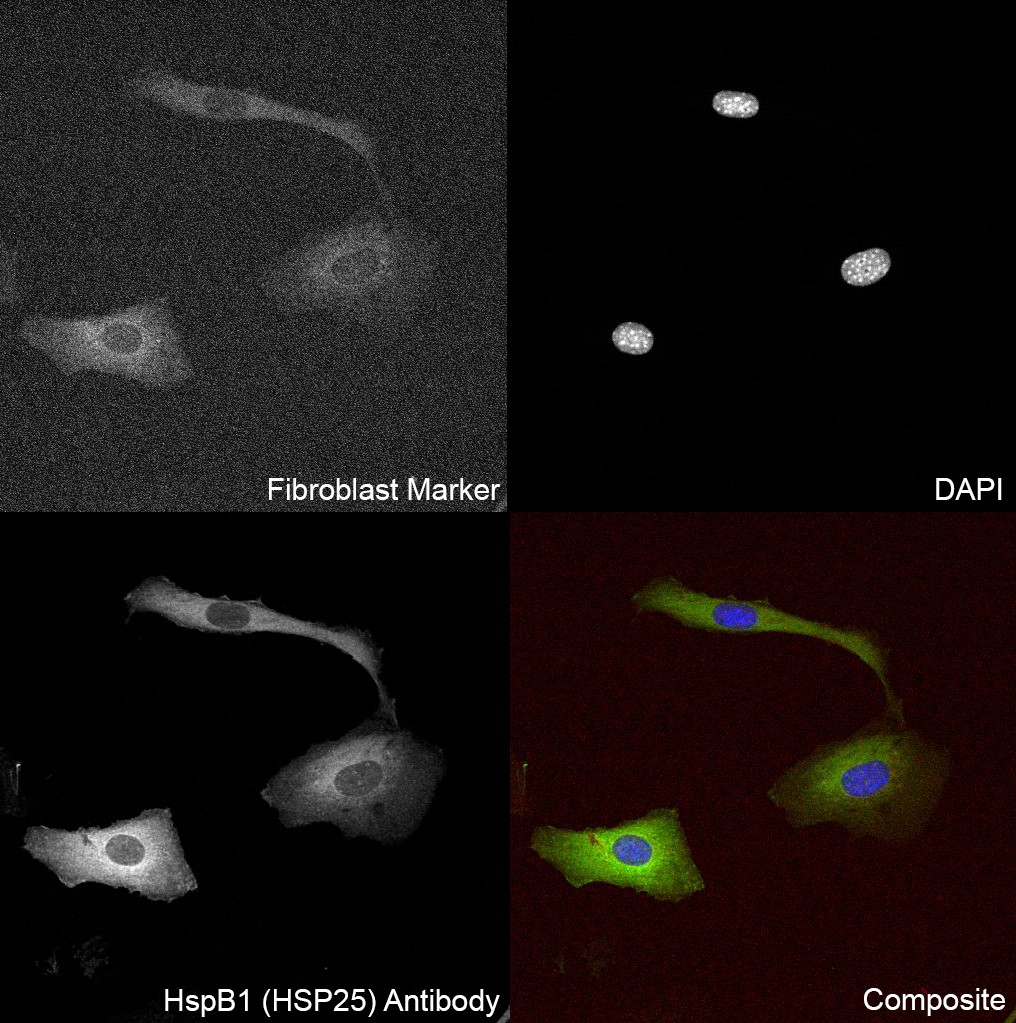

Supplement: glab296_suppl_Supplementary_Figure_2 [file glab296_suppl_supplementary_figure_2.jpeg]

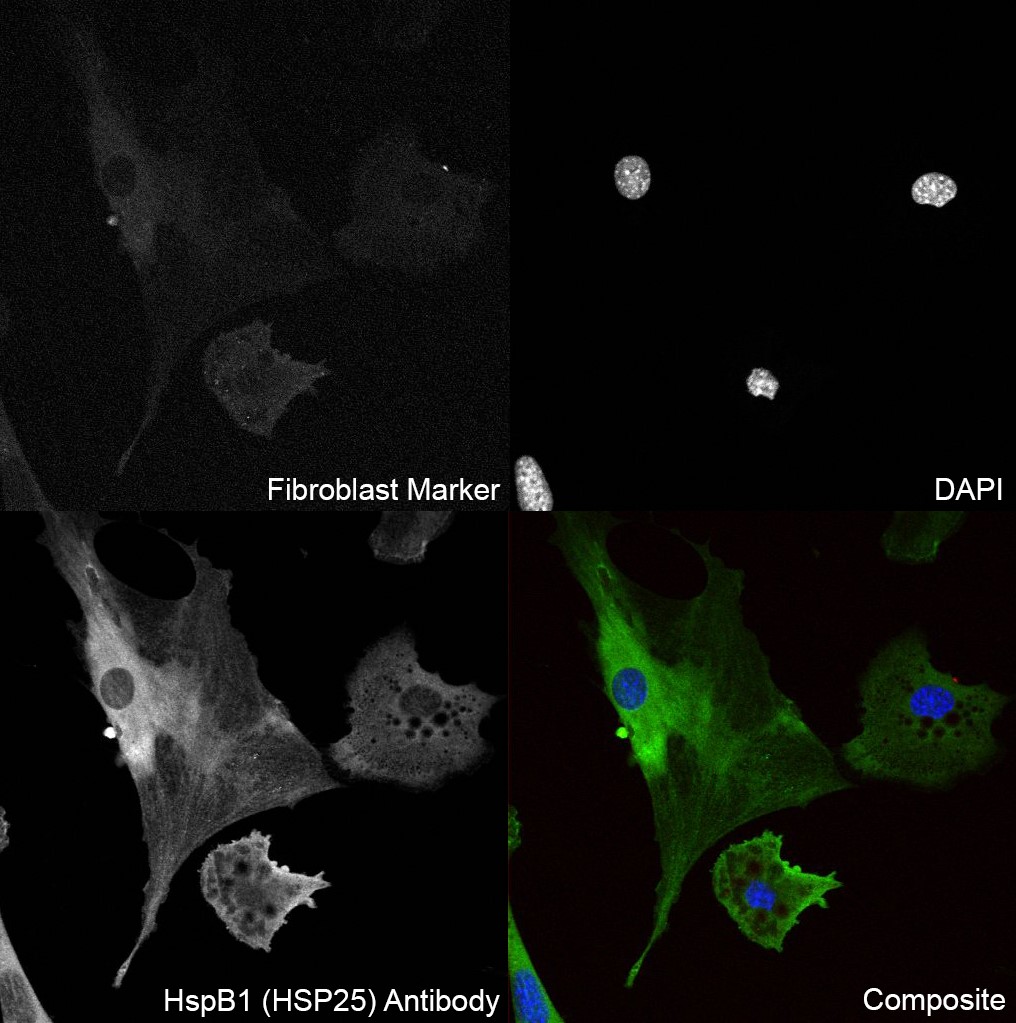

Supplement: glab296_suppl_Supplementary_Figure_3 [file glab296_suppl_supplementary_figure_3.jpeg]

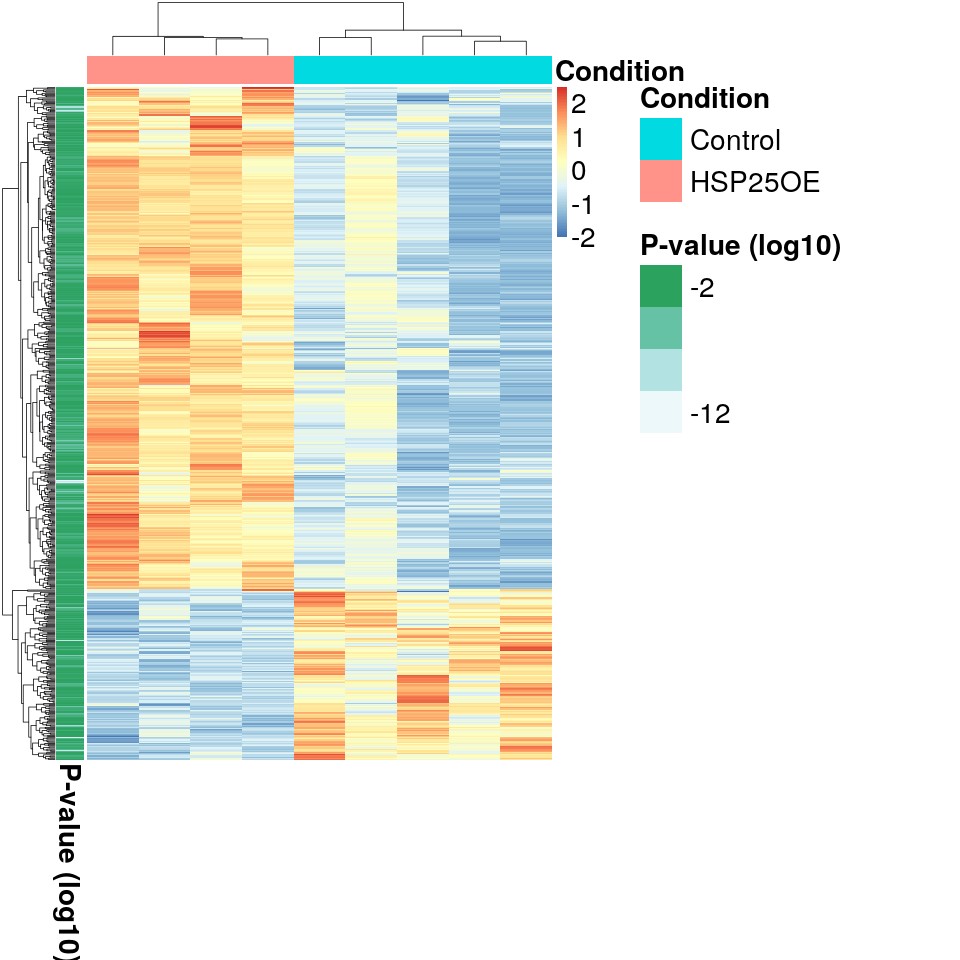

Supplement: glab296_suppl_Supplementary_Figure_4 [file glab296_suppl_supplementary_figure_4.jpeg]

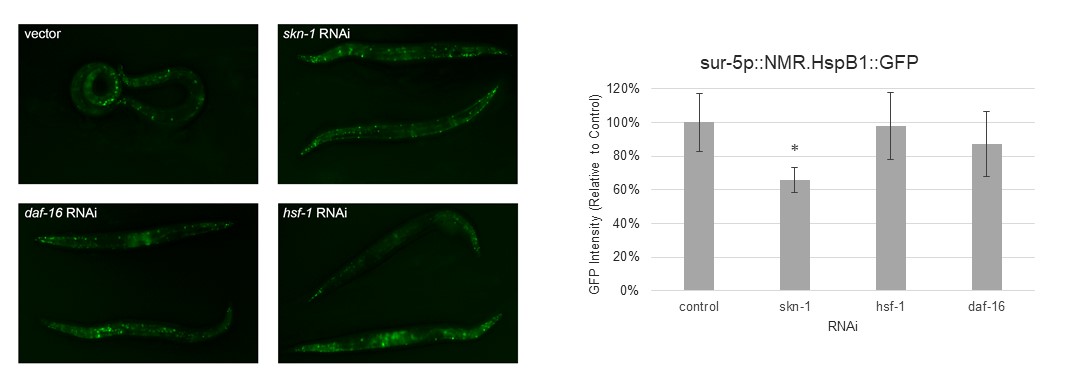

Supplement: glab296_suppl_Supplementary_Figure_5 [file glab296_suppl_supplementary_figure_5.jpeg]
